# Interaction of Gallium with a Copper Surface: Surface Alloying and Formation of Ordered Structures

**DOI:** 10.1021/acs.jpcc.3c05711

**Published:** 2023-10-11

**Authors:** Si Woo Lee, Arravind Subramanian, Fernando Buendia Zamudio, Jian Qiang Zhong, Sergey M. Kozlov, Shamil Shaikhutdinov, Beatriz Roldan Cuenya

**Affiliations:** † Department of Interface Science, Fritz Haber Institute of the Max Plank Society, Faradayweg 4-6, Berlin 14195, Germany; ‡ Department of Chemical and Biomolecular Engineering, National University of Singapore, 4 Engineering Drive 4, Singapore 117585, Singapore

## Abstract

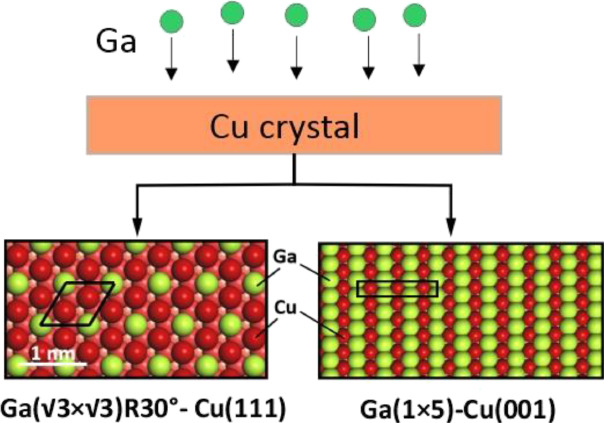

Alloys of gallium with transition metals have recently received considerable attention for their applications in microelectronics and catalysis. Here, we investigated the initial stages of the Ga–Cu alloy formation on Cu(111) and Cu(001) surfaces using scanning tunneling microscopy (STM), X-ray photoelectron spectroscopy (XPS), and low energy electron diffraction (LEED). The results show that Ga atoms deposited using physical vapor deposition readily intermix with the Cu surface, leading to a random distribution of the Ga and Cu atoms within the surface layer, on both terraces and monolayer-thick islands formed thereon. However, as the Ga coverage increases, several ordered structures are formed. The (√3×√3)*R*30° structure is found to be thermodynamically most stable on Cu(111). This structure remains after vacuum annealing at 600 K, independent of the initial Ga coverage (varied between 0.5 and 3 monolayers), indicating a self-limited growth of the Ga–Cu alloy layer, with the rest of the Ga atoms migrating into the Cu crystal. For Ga deposited on Cu(001), we observed a (1 × 5)-reconstructed surface, which has never been observed for surface alloys on Cu(001). The experimental findings were rationalized on the basis of density functional theory (DFT) calculations, which provided structural models for the most stable surface Ga–Cu alloys on Cu(111) and Cu(001). The study sheds light on the complex interaction of Ga with transition metal surfaces and the interfaces formed thereon that will aid in a better understanding of surface alloying and chemical reactions on the Ga-based alloys.

## 1 Introduction

Gallium (Ga) and its alloys have a range of properties, such as low melting point, nontoxicity, and the ability to wet metal and oxide surfaces, which make them attractive materials for application in microelectronics.^1^ A large interest in the Ga alloys, in particular with Cu, is mostly driven by their potential use in low-temperature soldering.^2^ In addition, Ga-containing intermetallic compounds and alloys have recently received considerable attention in catalysis. Gallium was found to be the efficient promoter for Cu, Ni, and Pd catalysts in several industrially important catalytic processes.^3−9^ Further development of Ga-promoted catalysts requires a much better understanding of the surface structures formed on the Ga alloys, which are difficult to predict a priori using a complex bulk phase diagram.^10,11^ However, fundamental studies on the interaction of Ga with metals, in particular, the surface structures and interfaces formed, remain rare and somewhat contradictory.^12−14^ For example, the physical vapor deposition (PVD) of Ga (up to 15 Å in nominal thickness) onto a Ni(001) substrate kept at room temperature was described as a layer-by-layer growth of an amorphous Ga film.^13^ In contrast, PVD of Ga (about 20 Å in thickness) onto a polycrystalline Au foil at 300–330 K led to the formation of a surface alloy with an average composition of Au_7_Ga_3_.^12^ In principle, even for metals that are immiscible in the bulk, surface alloying may take place such that the deposited metal intermixes with the surface layers of the substrate rather than forms a sharp interface between two metals. The formation of solely surface alloys is thought to be associated with the easier relaxation of the strain caused by the “guest” atoms in the “host” metal in the near-surface region compared to that in the bulk.^15^ Therefore, the ultimate structure of the surface alloy is often the result of a delicate balance of several energy terms such as surface energy and deformation energy, etc. Accordingly, the adsorption and other functional properties of the bimetallic surfaces critically depend on their atomic structure.^16,17^

In this work, we discuss the interaction of Ga with Cu. The Ga–Cu phase diagram^10^ suggests that Ga primarily forms solid solutions with Cu, and only one intermetallic compound, i.e., CuGa_2_, has been experimentally identified so far. Here, we investigated the initial stages of Ga deposition onto Cu surfaces and the atomic structures of the surfaces formed. In particular, we addressed the question of whether the Ga–Cu interaction is structure-sensitive by comparing the Ga deposition on Cu(111) and Cu(001) single-crystal surfaces. Using scanning tunneling microscopy (STM), X-ray photoelectron spectroscopy (XPS), and low energy electron diffraction (LEED) methods, we found that the Ga atoms on both surfaces readily intermix with the Cu surface, even at room temperature. Moreover, we observed several ordered structures such as Ga(√3 × √3)*R*30°–Cu(111) and Ga(1 × 5)–Cu(001). The atomic structures of the surface alloys were identified by careful analysis of their thermodynamic stability and STM image simulation using density functional theory (DFT).

## 2 Methods and Materials

### 2.1 Experimental Section

The experiments were carried out in a UHV chamber equipped with XPS, LEED, and STM (all from Specs). The Cu(111) and (001) single crystals (from MaTeck GmbH) were mounted onto stainless steel sample holder plates having a hole for heating the crystals by electron bombardment from the backside by using a thoriated tungsten filament. The temperature was measured by a chromel–alumel thermocouple placed in the hole at the edge of the crystal. The Cu surfaces were cleaned by several cycles of Ar^+^ sputtering at room temperature and UHV annealing at 900 K until LEED patterns showed sharp diffraction spots and no impurities were detected by XPS. Gallium (Aldrich, 99.999%) was deposited onto the sample kept at room temperature using an electron-beam assisted evaporator (Focus EFM3) from a BN crucible placed in a Mo liner. XPS spectra were measured using a monochromatic Al K_α_ X-ray source (*h*ν = 1486.6 eV) and a hemispherical analyzer (Phoibos 150). Analysis of the XPS spectra was performed using commercial software (CasaXPS, version 2.3.19). STM images were obtained at room temperature by using electrochemically etched W tips. Analysis and image processing were performed with the open source software Gwyddion (version 2.5)*.*

### 2.2 Computational Details

The DFT calculations were performed using the VASP package^18−20^ employing the PBE functional^21^ due to its high accuracy in the description of bulk properties of transition metals.^22^ The calculations on the Ga/Cu(111) surfaces were performed using (√3 × √3) slabs. For the Ga/Cu(001) surfaces, we used (2 × 2) and (1 × 5) slabs. For all systems, the slab was composed of six layers with all layers being allowed to relax. A *k*-mesh grid of (12 × 12 × 1) was used for all Cu(111) slabs and also for the (2 × 2) slabs of the Cu(001) surface, whereas a (20 × 3 × 1) mesh was used for the (1 × 5) slabs of Cu(001). Calculations were performed using the projector augmented wave (PAW) treatment of core electrons,^19^ the plane-wave basis set with the cutoff energy of 400 eV for the valence electrons, the Methfessel–Paxton smearing method with the smearing width of 0.1 eV, and electronic self-consistent convergence criteria of 1 × 10^–5^ eV. The criterion for geometry optimization was set to 0.03 eV·Å^–1^. The Bader charge analysis of the most stable systems was carried out employing the package developed by Henkelman et al.^23^ Simulation of the STM images of the optimized surface structures was performed based on the Tersoff and Hamman approach^24^ in the energy range of 0–0.2 V below the Fermi level.

## 3 Results and Discussion

### 3.1 Ga Deposition on Cu(111)

**Figure 1:**
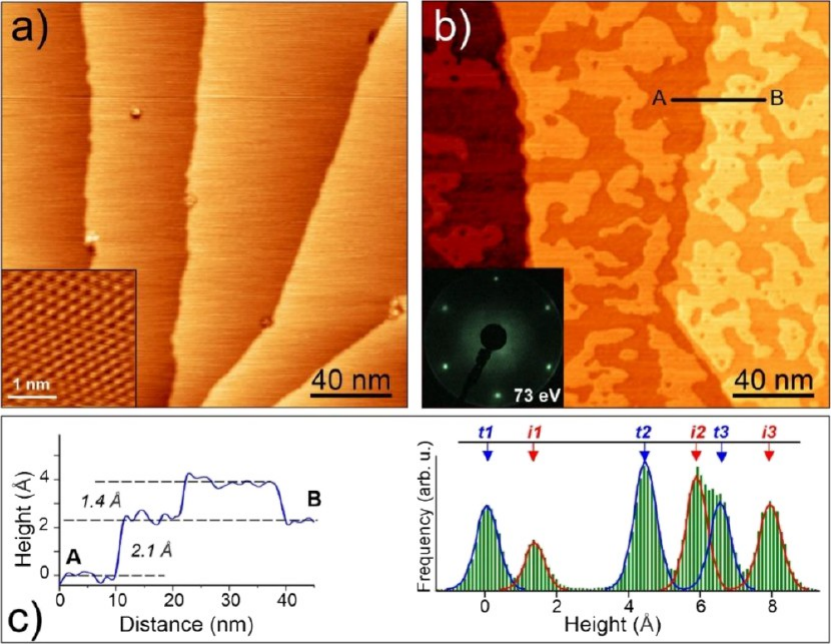
STM images of the Cu(111) surface before (a) and after (b) Ga deposition at room temperature (the Ga:Cu ratio is 0.16). Inset in (a) shows the atomically resolved image. Inset in (b) shows the corresponding LEED pattern (at 73 eV). (c) On the left, the profile line along (A, B) marked in the image (b); on the right, the height histogram of image (b), where *t*_*n*_ and *i*_*n*_ denote the *n*-th terrace and the islands thereon, respectively. Tunneling parameters: (a) sample bias 0.5 V, current 1 nA; 0.2 V, 4 nA (inset); (b) 1.4 V, 0.2 nA.

Figure 1a displays the typical morphology of the clean Cu(111) surface exposing wide and atomically flat terraces separated by monatomic steps. The high-resolution image shown in the inset was used for the STM calibration and azimuthal orientation of the crystal surface. To control the amount of Ga deposited, we used the Ga 2p:Cu 2p signal ratio in the XPS spectra measured at normal emission and normalized by using the well-known atomic sensitivity factors to obtain the Ga:Cu atomic ratio in the surface region probed by XPS, i.e., about 1.1 nm in thickness.^25^ Henceforth, this ratio will be used throughout the following discussion. Deposition of Ga at low coverages (the Ga:Cu ratio is 0.1–0.2) resulted in randomly distributed, irregularly shaped islands (Figure 1b), indicating the formation of kinetically limited structures at room temperature. Note also that the STM images showed no preferential nucleation at the step edges. Although the islands are sufficiently large in lateral size, the LEED patterns only showed the sharp (1 × 1) diffraction spots as on the pristine Cu(111) surface (inset in Figure 1b), indicating that the atoms within the islands are arranged in perfect registry with the atoms underneath. Based on these STM images, one would conclude that the islands are formed by the Ga adatoms. Indeed, the height of the islands (∼1.4 Å) is considerably smaller than the step height between the terraces (∼2.1 Å), as found by analysis of the line profiles and (more accurately) of the height histogram (Figure 1c). Although the height of nano-objects measured by STM depends on the local density of electron states and hence may deviate from their geometrical height, this finding at least indicates that the islands and terraces are considerably different with respect to their chemical compositions.

**Figure 2:**
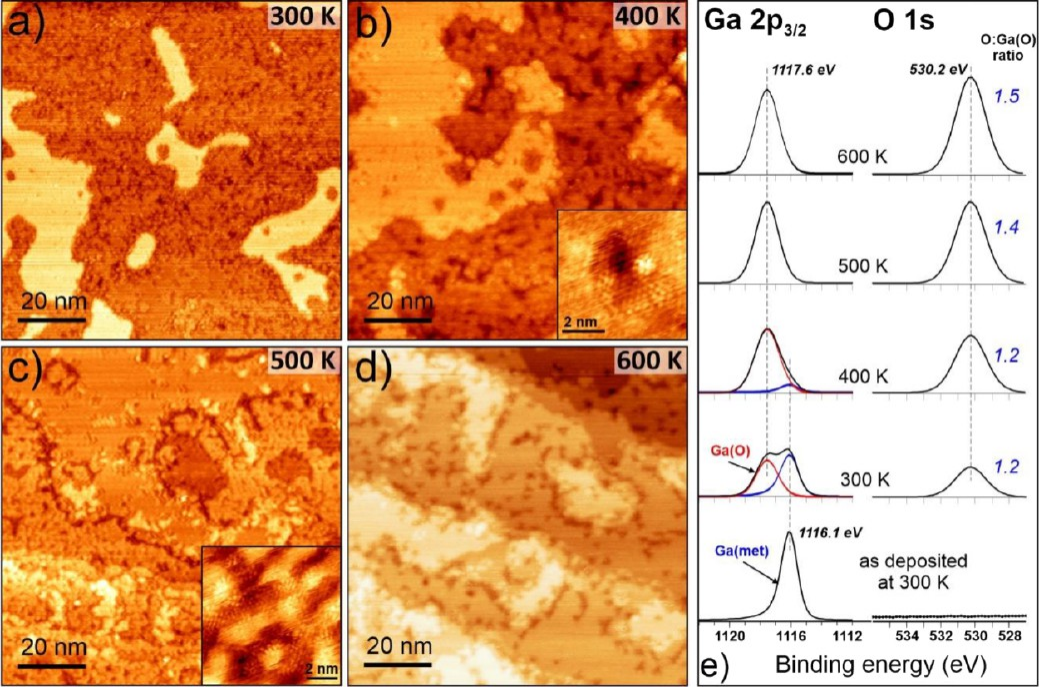
(a–d) STM images of the Ga/Cu(111) surface, shown in Figure 1b, after sequential exposure to 10^–6^ mbar of O_2_ for 15 min at temperatures increased stepwise from 300 to 600 K as indicated. The inset in (b) zooms in the “pits” randomly appearing on the flat islands observed in the large-scale images (a) and (b). The inset in (c) shows a typical high-resolution image of the more corrugated regions. (Tunneling parameters: (a) 1.0 V, 0.2 nA; (b) 1.0 V, 0.2 nA; (c) 1.0 V, 0.2 nA; (d) 1.0 V, 0.2 nA). Panel (e) shows Ga 2p_3/2_ and O 1s XPS spectra of the same sample before and after oxygen exposure at the indicated temperatures, all measured in UHV at 300 K. Blue and red lines show, respectively, metallic and oxidized states of Ga obtained by deconvolution. The atomic ratios of the O and Ga(O) species are shown adjacent to the O 1s spectra.

To gain more insight into the spatial distribution of the Ga atoms on the surface, we investigated the same sample after exposure to oxygen. We anticipated that Ga, which is well-known to be prone to oxidation, will manifest itself via the formation of oxidized Ga species with a different image contrast in STM. The results of these experiments are summarized in Figure 2, which shows STM images and corresponding XPS spectra after sequential exposures to 10^–6^ mbar of O_2_ for 15 min at each temperature increased stepwise from 300 to 600 K.

Not surprisingly, Ga is considerably oxidized even after O_2_ exposure at room temperature (Figure 2e). It is however remarkable that the ad-islands, which were thought to consist primarily of the deposited Ga atoms, remain almost intact, whereas the terraces become highly corrugated (Figure 2a). The oxidation of Ga is almost complete at 400 K, which is accompanied by further morphological changes on the terraces, assigned to oxidation of the Ga atoms therein. Meanwhile, the islands start to exhibit small dark spots (“pits”) (Figure 2b), which can be associated with oxidized Ga species, but can also be attributed to the initial stage of Cu oxidation.^26,27^ The oxidation of the clean Cu(111) surface at these pressure and temperature conditions usually results in complex Cu_2_O monolayer structures.^28,29^ Its formation does not show a measurable difference in the Cu 2p and Cu LMM Auger spectra,^30^ (see also Figure S1 in the Supporting Information (SI)) and only the O 1s signal at 529.6 eV shows up. The latter, however, overlaps with the state at 530.2 eV dominating the O 1s spectra on the Ga/Cu(111) surface and is assigned to Ga–O species (Figure 2e). Further oxygen treatments at 500 and 600 K do not alter the oxidation state of Ga but lead to a slight enrichment with oxygen as evidenced by the increase of the O:Ga(O) ratio with increasing oxidation temperature. This increase can indeed be explained by additional oxygen chemisorption on Cu. Overall, the morphological changes observed by STM can be described in terms of Ga oxidation and subsequent phase separation, ultimately resulting in Ga-oxide domains surrounded by the O/Cu(111) surface.

The results of the Ga titration experiments using O_2_ indicate that the islands, initially formed upon Ga deposition at submonolayer coverages, are actually enriched with Cu rather than being formed of the Ga adatoms. Accordingly, the terraces may contain most of the Ga atoms deposited. The formation of the Ga-rich terraces and Cu-rich islands can, in principle, be explained by the place exchange mechanism^31^ between adsorbed Ga atoms and surface Cu atoms. The Cu adatoms released upon this exchange can diffuse across the surface and aggregate into the islands but can also trap the incoming Ga adatoms. Therefore, the resulting surface morphology may depend not only on the substrate temperature but also on the Ga deposition flux (not varied here). Nonetheless, it is clear that Ga readily intermixes with the Cu(111) surface during deposition at room temperature. Such an intermixing has also been observed in our previous work on Ga deposited on Cu(001).^32^ Therefore, we can conclude that Ga and Cu intermixing at the surface is a structure-insensitive process.

**Figure 3:**
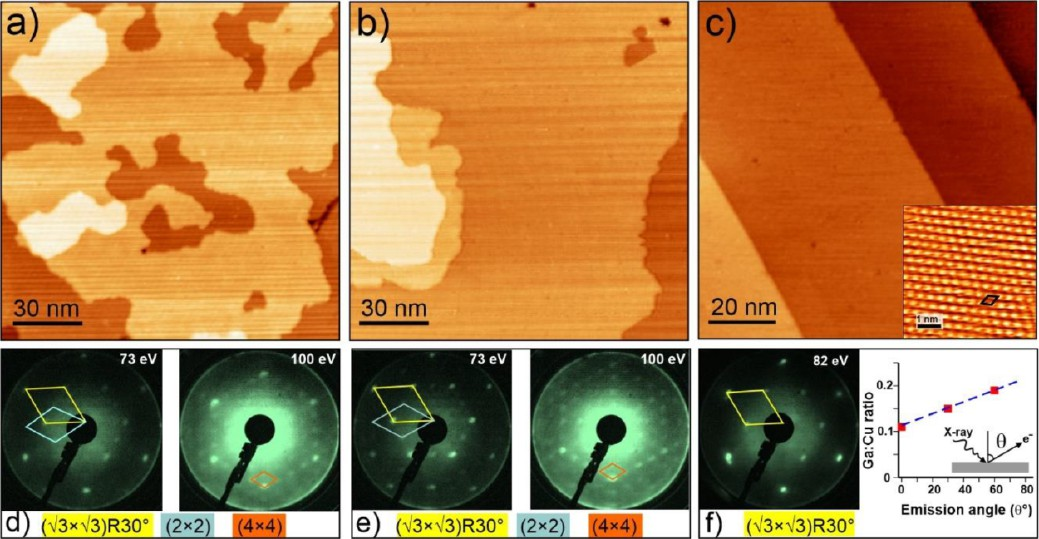
STM images (a, b) and corresponding LEED patterns (d, e) of the Cu(111) surface after Ga deposition at 300 K. The Ga:Cu ratios were 0.4 (a) and 1.0 (b), respectively. The unit cells determined by LEED and their notation below the patterns are shown in different colors. (c) Typical STM image of the Ga/Cu(111) surface after annealing in UHV at 600 K. Independent of the initial amount of Ga deposited (the Ga:Cu ratio between 0.1 and 1.0), atomically flat terraces showed the (√3×√3)*R*30° structure (f; inset in c). Tunneling parameters: (a) 1.0 V, 0.2 nA; (b) 1.0 V, 0.2 nA; (c) 0.3 V, 1 nA; 0.1 V, 7 nA (inset). Panel (f) also shows the Ga:Cu ratio measured by XPS as a function of the emission angle with respect to the surface normal.

Figure 3a,b displays STM images of Cu(111) at higher Ga coverages obtained by increasing the deposition time. Compared to the previous “low-coverage” sample (where the Ga:Cu ratio was 0.16, Figure 1b), the monolayer islands formed at Ga:Cu ratios of 0.4 (Figure 3a) and 1.0 (Figure 3b) are considerably larger, so it is difficult to differentiate original and newly formed terraces, all showing irregularly shaped edges. LEED inspection of the high-coverage samples revealed (2 × 2)-, (4 × 4)-, and (√3 × √3)*R*30°-Cu(111) ordered structures coexisting (Figure 3d,e).

Since the Ga deposition at room temperature may result in kinetically limited and hence metastable structures, the “as deposited” samples were annealed in UHV at 600 K for 15 min to facilitate Ga–Cu intermixing. STM images of the annealed surfaces (Figure 3c) only showed atomically flat wide terraces with well-oriented step edges. Both, LEED patterns and high-resolution STM images revealed solely the (√3×√3)*R*30° structure (Figure 3f, inset in Figure 3c), suggesting that it is thermodynamically the most stable. Interestingly, the annealed surfaces showed a Ga–Cu ratio of ∼0.1, irrespective of the initial amount of Ga deposited. Since the annealing temperature (600 K) is too low for the Ga sublimation to occur (indeed, the Ga vapor pressure at 600 K is below 10^–15^ mbar^33^), we conclude that the rest of the deposited Ga atoms migrated into the crystal bulk up to distances larger than the escape depth of the Ga 2p photoelectrons (∼1 nm).^25^ As shown in Figure 3f, the Ga:Cu XPS ratio in the annealed samples increases with increasing emission angle (with respect to the normal), suggesting that Ga is primarily located in the topmost surface layers. It therefore appears that the Cu(111) surface cannot accommodate more Ga atoms at elevated temperatures, thus indicating a self-limited formation of the Ga–Cu surface alloy.

### 3.2 Ga Deposition on Cu(001)

The initial stages of Ga deposition onto a Cu(001) substrate have been addressed in our previous study^32^ that revealed intermixing of Ga with the Cu(001) surface at room temperature. This conclusion was drawn also on the basis of the results of “titration” experiments with oxygen performed in the same manner as for Ga/Cu(111) discussed above. Here, after finding several ordered structures on the Ga/Cu(111) surface, we revisited the Ga/Cu(001) system by focusing on the surface alloy ordering.

**Figure 4:**
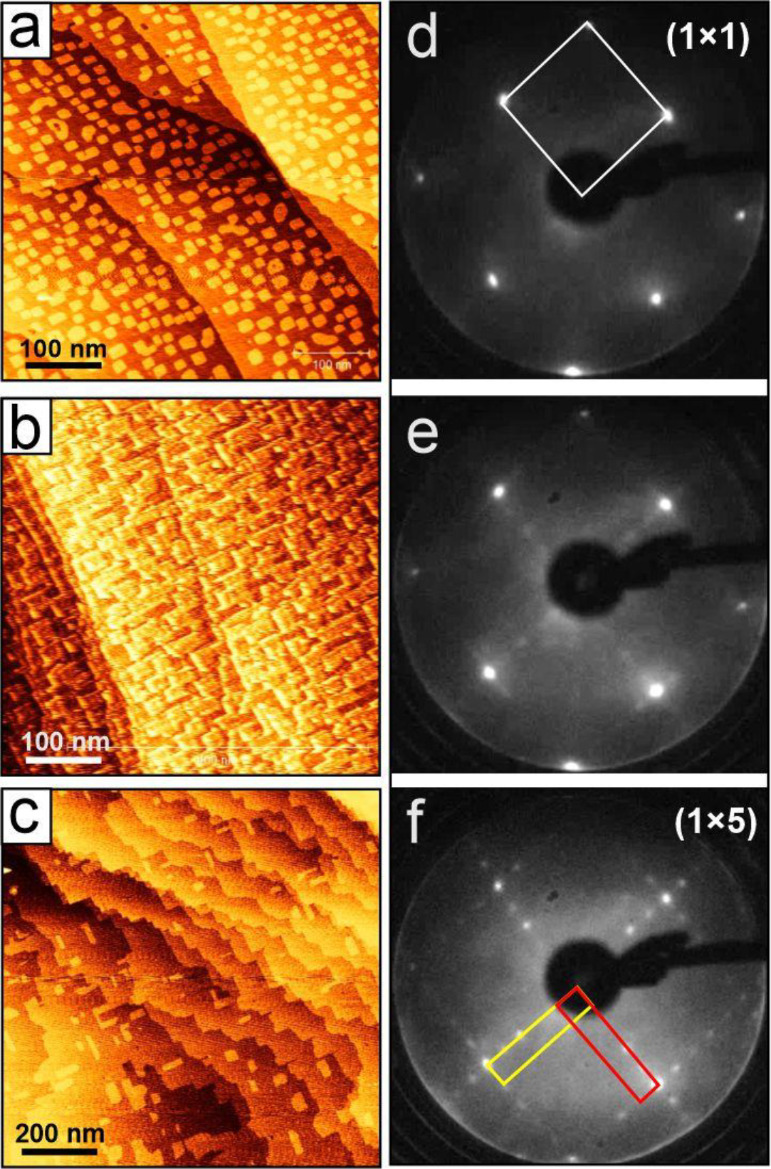
Large scale STM images (a–c) and corresponding LEED patterns (d–f) of the Ga/Cu(001) surfaces at increasing Ga coverage (from the top to bottom). All LEED patterns are obtained at 120 eV. The (1 × 5) unit cells for two rotational domains are shown in (f). Tunneling parameters: (a) 1.5 V, 0.08 nA; (b) 1.0 V, 0.2 nA; and (c) 0.7 V, 0.2 nA.

**Figure 5:**
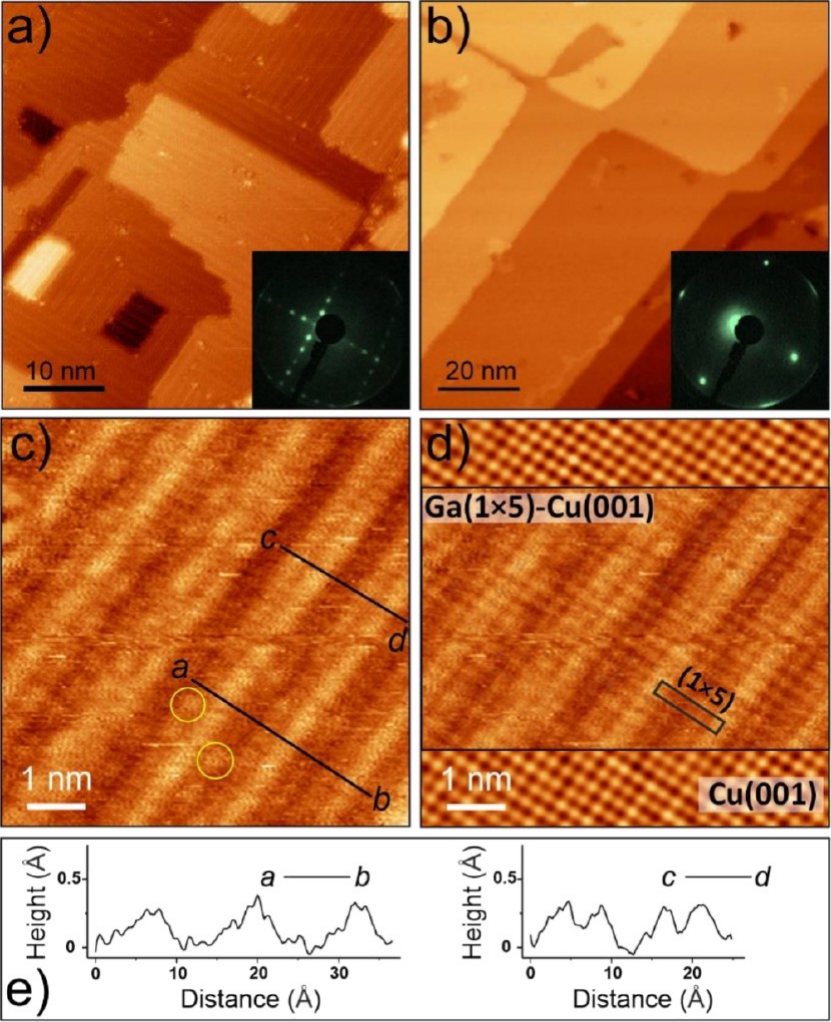
(a,b) STM images of the Ga(1 × 5)-Cu(001) surfaces: “as deposited” (a) and after UHV annealing at 600 K for 15 min (b). The corresponding LEED patterns (at 50 eV) are shown in the insets. (c) High-resolution STM image of the “as deposited” surface. The circles highlight some of the isolated protrusions appearing on either side of the protruding row. (d) Superposition of the image (c) and the image obtained on the clean Cu(001) surface prior to the Ga deposition in the transparent mode. (e) Topography profiles measured along the lines a-b and c-d in the STM image (c). Tunneling parameters: (a) 0.3 V, 0.2 nA; (b) 0.3 V, 0.2 nA; (c) 0.2 V, 6 nA; (d) 0.2 V, 7 nA for pristine Cu(001).

Figure 4 shows large-scale STM images and LEED patterns of the Ga/Cu(001) surface at increasing amounts of Ga deposited at 300 K. At low Ga coverages, numerous square islands are formed, with the edges running along the main crystallographic orientations of the Cu(001) surface (Figure 4a). Note, however, that as in the case of Ga/Cu(111), the Ga coverage cannot be determined by STM due to the Ga–Cu intermixing. LEED patterns keep showing only (1 × 1) spots of Cu(001) (Figure 4d), suggesting a rather random distribution of the Ga atoms at low coverages. With increasing Ga coverage, the islands coalesce (Figure 4b), and LEED starts to show additional weak spots (Figure 4e) which develop into a clear (1 × 5)-Cu(001) pattern at further increasing coverage (Figure 4f, see also the inset in Figure 5a). For the latter sample, only relatively wide terraces with a few rectangular islands are observed (Figure 4c).

High-resolution STM images of the (1 × 5)-Cu(001) surface (Figure 5a) revealed two rotational domains, each showing slightly protruding atomic rows running along the crystallographic directions of the Cu(001) surface. The average distance between these rows measured by STM (13 Å) agrees with the value of 5 × 2.55 Å = 12.75 Å expected for the (1 × 5) superstructure. The apparent height modulation measured across the rows is about 0.3 Å (Figure 5e), pointing to a slight buckling of the surface layer, although electronic effects may also contribute to the STM image contrast. Indeed, the atomic protrusions observed in high-resolution STM images are considerably larger than those imaged on the clean Cu(001) surface at similar tunneling conditions, as shown in Figure 5d.

The STM image in Figure 5c also shows that the Ga(1 × 5)–Cu(001) surface formed at room temperature is not perfectly ordered on the atomic scale. In an attempt to improve the surface ordering, the sample was heated in UHV and monitored by LEED. It turned out that the initially sharp (1 × 5) diffraction spots (inset in Figure 5a) only attenuated on heating and finally disappeared after 15 min of annealing at 600 K, while the (1 × 1) spots remained (inset in Figure 5b). The corresponding STM images showed atomically flat, wide terraces as on the pristine Cu(001) surface. Concomitantly, the Ga:Cu ratio is substantially decreased, i.e., from 0.3 to 0.08, indicating considerable Ga migration into the Cu crystal bulk upon heating to elevated temperatures. The Ga atoms that remain at the annealed Cu surface seem to be randomly distributed at the surface.

Even further increasing the Ga coverage causes the (1 × 5) LEED pattern to attenuate. The corresponding STM images revealed the formation of large, sticklike deposits on top of the flat terraces (see Figure S2 in the SI). However, subsequent UHV annealing of this sample at 700 K caused the disappearance of these structural features and restoration of the sharp (1 × 5) pattern. We can therefore conclude that the (1 × 5) structure only forms at a particular Ga surface coverage tuned either by deposition directly or by subsequent heating.

### 3.3 DFT Analysis of Ordered Structures on Ga/Cu(111)

**Figure 6:**
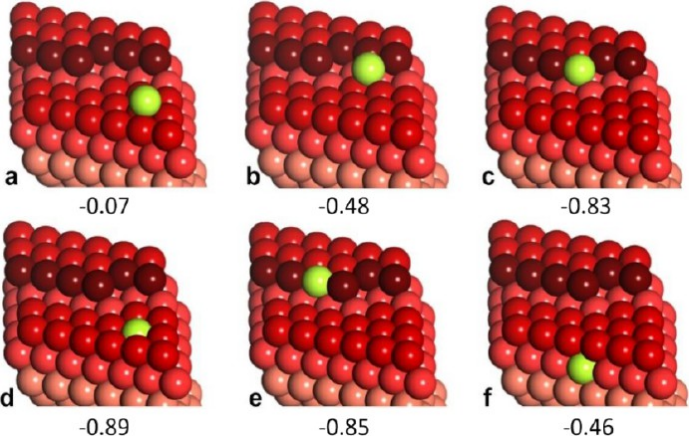
Atomic models (in perspective view) and adsorption energies (in eV) calculated for a Ga atom on the Cu(27 35 37) surface (details in the text). Color code: Ga atom (green), Cu step atoms (dark red), Cu atoms in the (111) layers are in different shades of red: the deeper the layer, the lighter its shade.

We first addressed the adsorption of a single Ga atom on Cu(111). In the following calculations, we used a slab exposing the Cu(27 35 37) surface (Figure 6) in order to calculate adsorption energies not only on the regular (111) terraces but also on defect sites such as steps and kinks. The adsorption energy was defined as
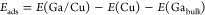
where *E*(Ga/Cu) and *E*(Cu) are the total energies of the slabs with and without a Ga atom, respectively, and *E*(Ga_bulk_) is the energy of a Ga atom in the Ga single crystal.

Among the adsorption sites on the (111) terrace, Ga adsorption in the 3-fold hollow site is found to be the most stable (*E*_ads_ = −0.07 eV, Figure 6a). Not surprisingly, adsorption at the step edge is much stronger (*E*_ads_ = −0.48 eV, Figure 6b). Adsorption of Ga at the kink site is even stronger (*E*_ads_ = −0.83 eV, Figure 6c), pointing to preferential nucleation of Ga at the kink if the Ga adatoms readily diffuse across the terrace. We also considered the place exchange mechanism , where a Ga adatom on the (111) terrace replaces a Cu atom underneath. In our model, the Cu atom released by this exchange diffuses at the surface and ultimately adsorbs at the kink site (Figure 6d). The corresponding net adsorption energy is even higher than that for the Ga atom directly adsorbed at the kink site (−0.89 vs −0.83 eV). The results suggest the place exchange adsorption mechanism on terraces as the most favorable. Moreover, such a scenario may also be applied to a Ga atom that first adsorbs at the step but ultimately becomes incorporated into the step edge (*E*_ads_ = −0.85 eV, Figure 6e). Relatively low stability of a Ga single atom in the subsurface layer (*E*_ads_ = −0.46 eV, Figure 6f) hinders its further diffusion into the crystal bulk, thus stabilizing Ga atoms on the surface. Therefore, the DFT results provide a solid rationale for an easy intermixing of the Ga and Cu atoms within the surface layer during Ga deposition.

In the next step, we analyzed the thermodynamic stability of ordered Cu_*n*_Ga_*m*_ overlayers on Cu(111), where *n* and *m* denote the numbers of Cu and Ga atoms in the (√3 × √3)*R*30°-Cu(111) unit cell, respectively. Since the relative stability of the surface depends on the amounts of Ga and Cu at the surface, we calculated the formation energy of the alloy surface (γ_form_) as a function of the chemical potentials of Cu and Ga (μ_Cu_ and μ_Ga_) as
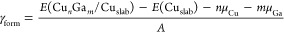
where *A* is the surface area, and *E*(Cu_*n*_Ga_*m*_/Cu_slab_) and *E*(Cu_slab_) are the total energies of a Cu(111) slab with and without the Cu–Ga overlayer, respectively. The μ_Cu_ and μ_Ga_ values were referenced to the energies of the Cu and Ga atoms in the pure bulk, i.e., μ_Cu_ = μ(Cu_bulk_) and μ_Ga_ = μ(Ga_bulk_) + Δμ_Ga_.

**Figure 7:**
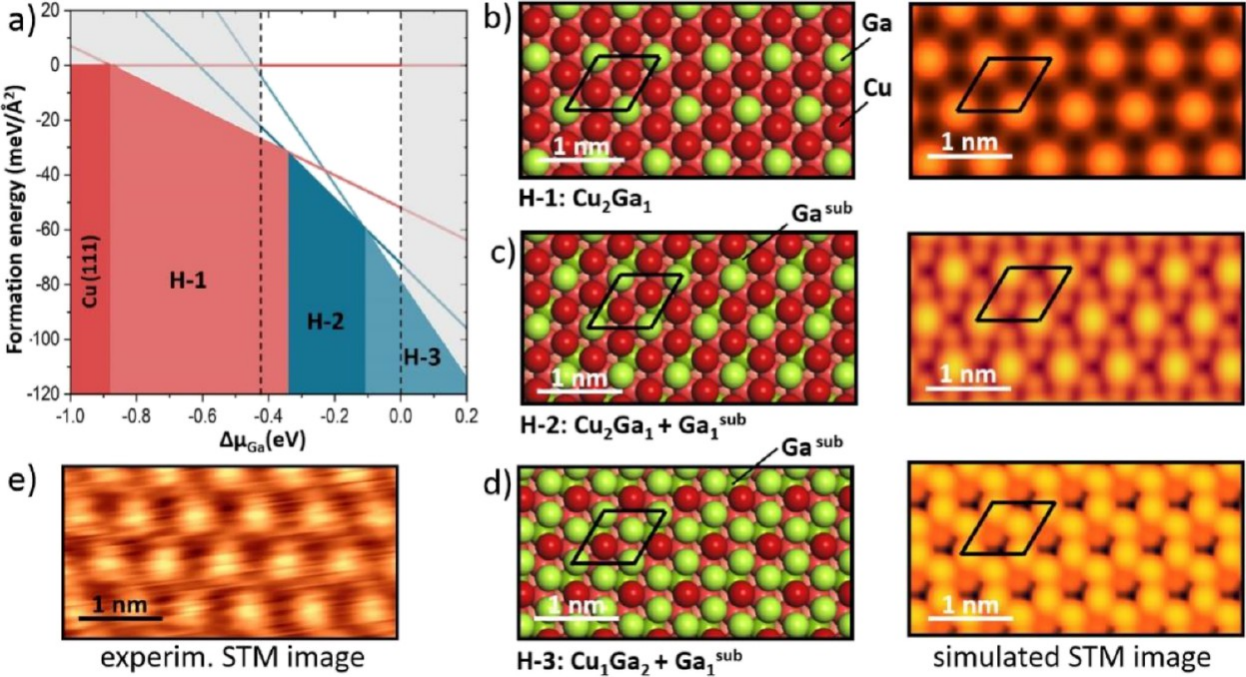
(a) Surface phase diagram calculated for the Ga–Cu mixed layers exhibiting a (√3 × √3)*R*30°-Cu(111) symmetry as a function of the Ga chemical potential. Dashed lines indicate the experimentally feasible range of chemical potentials. (b–d) Top views of the model structures (Ga, green; Cu, red) and the corresponding simulated STM images. The (√3 × √3)*R*30° unit cell is indicated. Structure H-1 has Ga atoms only in the surface layer, while H-2 and H-3 have additional Ga atoms in the subsurface layer (Ga^sub^). For the STM simulation, the electron states in the range of 0–0.2 V below the Fermi level were considered. For comparison, the experimental STM image recorded at 0.1 V is shown in (e).

Figure 7a displays a surface phase diagram with the most stable structures obtained by calculations of all possible Cu_*n*_Ga_*m*_ (*n* = 0–3, *m* = 0–3, *n* + *m* ≤ 3) mixed layers, including the structures having Ga atoms in the subsurface layer (Ga^sub^) (see Figure 7b–d and Table S1 in the SI). Structure H-1 describes a surface layer that is composed of two Cu atoms and one Ga atom in the surface unit cell (denoted Cu_2_Ga_1_). Structures H-2 and H-3 correspond to Cu_2_Ga_1_ and Cu_1_Ga_2_ surface layers, having an additional Ga^sub^ atom in the subsurface layer. The dashed lines in Figure 7a indicate the experimentally feasible range of chemical potentials that vary between the chemical potentials of Ga atoms in pure Ga bulk (Δμ_Ga_ = 0 eV) and in the Cu-rich Cu–Ga alloys, in this case, Cu_7_Ga_1_ (Δμ_Ga_ = −0.43 eV).

Among the structures considered, the simulated STM image of the Cu_2_Ga_1_ (H-1) surface best matches the experimentally observed one shown in Figure 7e for direct comparison. Accordingly, the protrusions seen in the STM images correspond to the positions of the surface Ga atoms. The Bader charge analysis of this Cu_2_Ga_1_ structure (Table S1 in the SI) indicates a charge transfer (0.20 *e*^–^) from Ga to Cu atoms, which is close to 0.18 *e*^–^ calculated for Ga in the bulk of the Cu_7_Ga_1_ alloy.

### 3.4 DFT Analysis of Ordered Structures on Ga/Cu(001)

**Figure 8:**
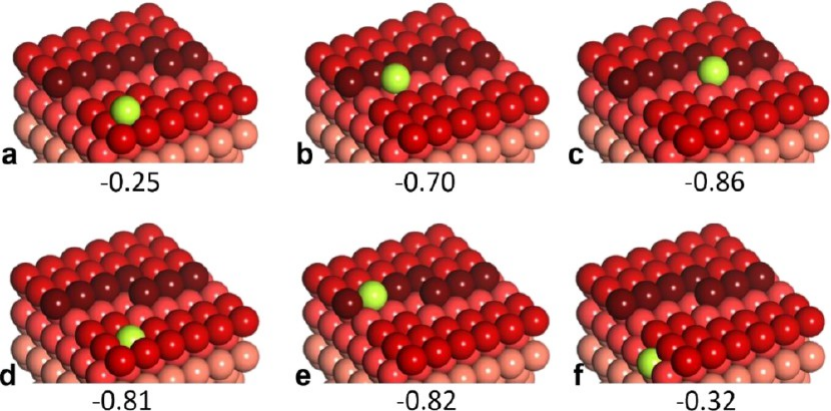
Atomic models (in perspective view) and corresponding adsorption energies (in eV) calculated for a Ga atom on the Cu(3 5 35) surface (details in the text). Color code: Ga atom (green), Cu step atoms (dark red), Cu atoms in the (001) layers are in shades of red: the deeper the layer, the lighter its shade.

Within the same approach employed for the Ga/Cu(111) system, the adsorption of a single Ga atom on Cu(001) was studied using a high Miller index slab, in this case, Cu(3 5 35), which exposes the (001) terrace and also a step and kink as one of the possible adsorption sites (Figure 8). Among regular adsorption sites for a Ga adatom on the Cu(001) surface, the 4-fold hollow site is the most favorable (*E*_ads_ = −0.25 eV, Figure 8a). As expected, adsorption on the step edge is much stronger (*E*_ads_ = −0.70 eV, Figure 8b) and even stronger at the kink site (*E*_ads_ = −0.86 eV, Figure 8c). Place exchange with a Cu atom on the terrace, which in turn migrates to the kink site, results in a net adsorption energy of −0.81 eV (Figure 8d), which is almost the same as for the Ga atom incorporated into the step edge (−0.82 eV, Figure 8e). Migration of the surface Ga atom into the subsurface layers is thermodynamically unfavorable (*E*_ads_ = −0.32 eV, Figure 8f).

Therefore, the DFT results revealed, in essence, no significant differences in the behavior of a Ga adatom on the Cu(111) and Cu(001) surfaces. Although the Ga atom initially binds to the (001) surface considerably stronger than on the (111) surface (*E*_ads_ are −0.25 and −0.07 eV, respectively), the place exchange mechanism dominates the adsorption process, leading to the facile Ga–Cu intermixing within the surface layer on both systems, in nice agreement with the experimental results.

**Figure 9:**
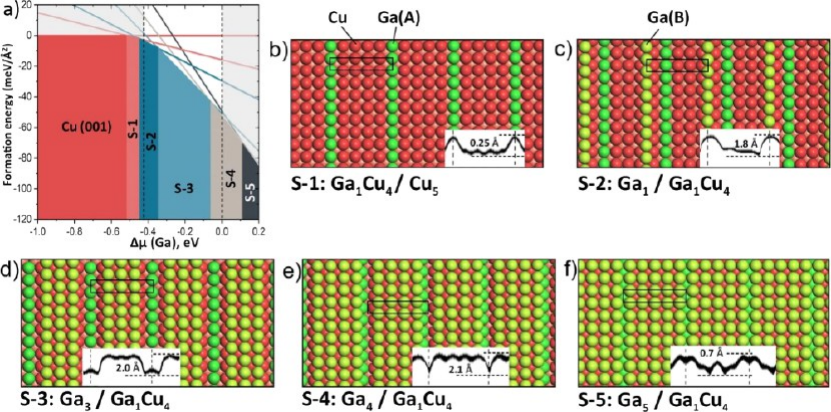
(a) DFT-derived surface phase diagram obtained for Ga_*n*_Cu_*m*_ (*n* + *m* ≤ 5) overlayers showing the (1 × 5)-Cu(001) symmetry. Dashed lines indicate the experimentally feasible range of the Ga chemical potentials. (b–f) Top views of the structures S-1–S-5 described in terms of “surface/sub-surface” layers in the (1 × 5) slab. Two differently coordinated Ga atoms in the unit cell are labeled Ga(A) and Ga(B), for clarity. The insets show the profile lines along the {100} direction obtained from simulated STM images (all shown in Figure S3). The apparent corrugation amplitudes are indicated (in Å).

To survey possible surface alloy structures exhibiting the (1 × 5)-Cu(001) symmetry, we calculated the formation energies of Cu_*n*_Ga_*m*_ surface layers (*n* = 0–5; *m* = 0–5; *n* + *m* ≤ 5) on a Cu(001)-(1 × 5) slab. In addition, we varied the composition of the subsurface layer by replacing some of the Cu atoms within the (1 × 5) unit cell with Ga. The formation energies calculated for about 140 different structures are summarized in Tables S2 and S3 in the SI, which reveal several structures having very close γ_form_ values. The phase diagram based on these calculations is shown in Figure 9a. However, the models denoted S-2, S-3, and S-4 in this diagram feature “missing row” or “added row” types of structures, which all exhibit the atomic corrugation amplitude of about 2 Å (see profile lines in insets in Figure 9c–e, and simulated STM images in Figure S3 in the SI), i.e., much higher than the experimentally measured value of 0.3 Å (Figure 5e). Therefore, our analysis should be focused on the “dense” Cu_*n*_Ga_*m*_ overlayers (i.e., *n* + *m* = 5). Indeed, the model S-1 denoting the Ga_1_Cu_4_ surface layer exhibits a corrugation of ∼0.25 Å (Figure 9b), i.e., very close to the value observed in the experiment. However, this structure possesses the lowest amount of Ga in the unit cell, whereas the (1 × 5)-reconstructed surface develops at high Ga coverages (Section 3.2). On the other hand, the high coverage model S-5 denoting the Ga_5_ surface layer with one additional Ga atom in the subsurface layer exhibited a corrugation of ∼0.7 Å (Figure 9f), which is significantly higher than 0.3 Å observed experimentally. The analysis of the numerous structures presented in Tables S2 and S3 could not find the one that would fit all of our selection criteria, e.g., thermodynamic stability at experimentally relevant chemical potentials of Ga, and good fit between experimental and simulated STM images. Moreover, our additional calculations of the “short-range” superstructures, such as (2 × 2) and *c*(2 × 2), never observed in our experiments but previously reported for Pd/Cu(001)^34,35^ and Mn/Cu(001),^36^ revealed that they are even more stable than the above-shown structures S1–S3 exhibiting the (1 × 5) symmetry (see Figure S4 in the SI). All of these findings prompted us to expand the range of possible (1 × 5) structures.

We recall that the atomic structure of a metal and oxide overlayer deposited onto another metal surface is often considered in terms of a coincidence structure (such as a Moiré pattern) that is formed between the overlayer and the substrate. Importantly, the surface layer in such systems is buckled in the range of a few tenths of Angstrom,^37^ i.e., the range observed in our system. Note also that a (*n* × *m*) type of reconstructions (with *m* ≫ *n*) is well documented for the clean (001) surfaces of noble metals, e.g., Au(001)-(5 × 20), Pt(001)-(5 × 20), and Ir(001)-(1 × 5), all showing a slightly buckled pseudohexagonal surface layer.^38−41^ To examine whether a similar structural motif can be applied to the Ga(1 × 5)-Cu(001) surface, we carried out calculations for a mixed Cu_*n*_Ga_6–*n*_ (*n* = 0–6) layer on top of the (1 × 5)-Cu(001) slab.

The atomic structures and corresponding formation energies are presented in Table S4 in the SI. The results show that pure Ga and Cu quasi-hexagonal layers are thermodynamically unstable (the corresponding energies are positive). Interestingly, the formation energy value goes through the minimum obtained for a Cu_3_Ga_3_ composition. Moreover, the corresponding energy is even lower than those calculated for all previous structures in this range of chemical potentials. The highest stability of the Cu_3_Ga_3_ structure is likely due to the highest number of Cu–Ga bonds per unit cell among other structures considered. The Bader charge analysis revealed that the Ga atoms in the surface layer experience a loss of electrons between 0.15 and 0.20 *e*^–^ (Table S4), which is similar to 0.18 *e*^–^ obtained by calculations of the Cu-rich bulk alloy with a Cu_7_Ga stoichiometry.

**Figure 10:**
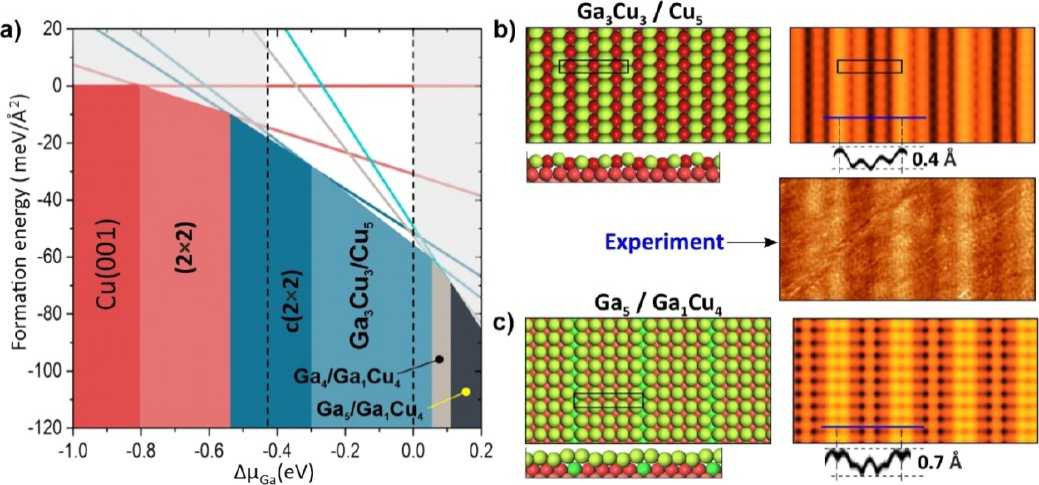
(a) Final phase diagram obtained from calculations of all structures considered so far for the Ga–Cu surface alloy on Cu(001). (b) DFT-optimized atomic model of the incommensurate Cu_3_Ga_3_ layer on Cu(001)-(1 × 5) is shown in the top and cross views (Ga—green, Cu—red). The simulated STM image for this structure and the profile line are also shown. (c) Atomic model, simulated STM image, and profile line obtained for the commensurate Ga_5_ monolayer with an additional Ga atom in the subsurface layer. The experimental image from Figure 5 is reproduced here for a direct comparison.

The final phase diagram combining the results obtained on all ordered structures computed so far is shown in Figure 10a. In this diagram, the (2 × 2) and *c*(2 × 2) structures are found to be the most stable at low chemical potentials (e.g., low Ga coverage). It should be noted that Ga atoms in these models are embedded into the surface layer (via the place exchange mechanism, see above) and are not adsorbed onto the Cu surface. The fact that these structures were not observed in our experiments can tentatively be explained by a relatively slow diffusion of Ga atoms within the surface layer at room temperature.

The incommensurate Cu_3_Ga_3_ layer placed on top of the Cu(001)-(1 × 1) surface resulting in the (1 × 5) coincidence superstructure (Figure 10b) appears to be the most favorable in the experimentally relevant range of Ga chemical potentials. STM images simulated for this structure (Figure 10b, see also Figure S5) also showed a fairly good match with the experimental image, as far as both the image contrast and the corrugation amplitude are concerned. Indeed, the simulation revealed surface rumpling of about 0.4 Å, which is comparable with 0.3 Å observed in the experiment. However, we cannot completely rule out the model of a Ga(001) monolayer with an additional Ga atom in the subsurface to induce (1 × 5) symmetry (Figure 9c), which was calculated to be stable at higher Ga chemical potentials.

## 4 General Discussion and Conclusions

The formation of ordered surface alloys on the Cu surfaces in the sub- and near-monolayer coverage regime has previously been reported for Cu(111),^42−44^ Cu(110),^45^ and Cu(001).^34,35,46^ The degree of ordering was found to depend not only on the nature of the deposited metal but also on the metal coverage and the temperature used either for deposition or postannealing. In the great majority of cases, metals deposited on Cu(111) showed a (√3 × √3)-*R*30° ordering. To the best of our knowledge, ordering on a longer range was only reported for the case of Se_2_(gas) deposition at room temperature. Although the Cu–Se alloy layer formed locally showed the (√3 × √3)-*R*30° structure, a long-range surface modulation was clearly observed by STM via a stripelike morphology. This particular structure was explained in terms of a slight distortion of the two-dimensional Cu–Se alloy layer along a particular orientation of Cu(111) underneath.^43^

For metals on the Cu(001) surface, the *c*(2 × 2) type of reconstruction dominates the structure. However, the *c*(2 × 10) superstructure was observed upon deposition of Ag at coverages close to a monolayer.^47^ This long-range ordered structure was assigned to the formation of a Ag(111)-like single layer (monolayer) on top of the unreconstructed Cu(001)-(1 × 1) surface.

Our combined experimental and theoretical studies of the initial stages of Ga physical vapor deposition onto the Cu(001) and Cu(111) single-crystal surfaces show that Ga atoms intermix with the Cu surface from the onset. The DFT results revealed no significant difference in the adsorption behavior of a Ga adatom on the Cu(111) and Cu(001) surfaces. In both cases, the place exchange mechanism dominates the adsorption process, leading to facile intermixing of the Ga and Cu atoms. Migration into the subsurface layers during the deposition is thermodynamically unfavorable. Therefore, the Ga atoms are primarily located within the surface layer. As the amount of deposited Ga increases, several ordered structures are formed, as observed by LEED and STM.

The (√3×√3)*R*30° structure is found to be the most stable on Cu(111) as this structure remains after vacuum annealing at 600 K. It therefore appears that Ga follows the general trend observed thus far for other metals deposited onto Cu(111), mostly showing the (√3 × √3)*R*30° reconstruction. Based on our DFT results, we attributed this structure to the surface layer with a Cu_2_Ga_1_ composition. Interestingly, this structure is formed after vacuum annealing independent of the initial Ga coverage (varied between 0.5 and 3 ML), thus indicating a “self-limited” growth of the Ga–Cu alloy at the surface, while the rest of the Ga atoms migrate into the Cu bulk, at least deeper than 1 nm probed by XPS. Such migration resulting in a nonuniform Ga distribution may have an impact on the functional properties of nanoparticulate bimetallic systems and even lead to particle size effects, for example, on the reactivity of metal nanoparticles formed in the Ga-promoted catalysts.

Upon Ga deposition onto Cu(001) at room temperature, we observed a previously unknown (1 × 5)-Cu(001) ordered structure that appears in a particular range of (relatively high) coverages before the growth of three-dimensional deposits sets in. Interestingly, if such a coverage was exceeded by deposition, the same surface reconstruction can be obtained by mild annealing in vacuum since this causes migration of the Ga atoms that are not involved in the surface alloy formation into the crystal bulk. We have calculated numerous atomic models for this surface but could not identify a structure explaining both thermodynamic stability and STM image contrast. Therefore, we turned to so-called “hexagonal” reconstructions, which are well-documented for clean noble metal surfaces, in particular, for Ir(001)-(1 × 5). Indeed, a quasi-hexagonal noncommensurate Cu_3_Ga_3_ overlayer consisting of six atoms placed over five Cu atoms in the (1 × 5)-Cu(100) unit cell revealed high thermodynamic stability and a fairly good fit between the simulated and experimental STM images.

Overall, our study sheds light on the complex interaction of Ga atoms with transition metal surfaces and the interfaces formed thereon, which is a prerequisite for a deeper understanding of the surface alloy formation and chemical reactions on the Ga-containing alloys.

## Supporting Information Available

XPS spectra of the Ga/Cu(111) surface; additional STM and LEED results for the Ga(1 × 5)-Cu(001) structure; atomic structures; STM image simulations and the formation energies calculated for numerous models of Ga/Cu(111) and Ga/Cu(001) ordered surfaces; and Bader charge analysis for the most stable structures (PDF)
