# Supplementary material for: Interaction of Gallium with a Copper Surface: Surface Alloying and Formation of Ordered Structures
Source: J Phys Chem C Nanomater Interfaces. 2023 Oct 11;127(42):20700–9. doi: 10.1021/acs.jpcc.3c05711 (PMC10614298; doi:10.1021/acs.jpcc.3c05711)

Supporting Information (SI)

Interaction of Gallium with a Copper Surface: Surface Alloying and Formation of Ordered Structures.

Si Woo Lee,[†1] Arravind Subramanian,[†2] Fernando Buendia Zamudio,[2] Jian Qiang Zhong,[1] Sergey Kozlov,[2*] Shamil Shaikhutdinov,[1*] Beatriz Roldan Cuenya[1]

[1] Department of Interface Science, Fritz Haber Institute of the Max Plank Society, Faradayweg 4-6, 14195 Berlin, Germany

[2] Department of Chemical and Biomolecular Engineering, National University of Singapore, 4 Engineering Drive 4, Singapore 117585, Singapore

*Corresponding authors: shaikhutdinov@fhi-berlin.mpg.de (S. Shaikhutdinov)
sergey.kozlov@nus.edu.sg (S. Kozlov)

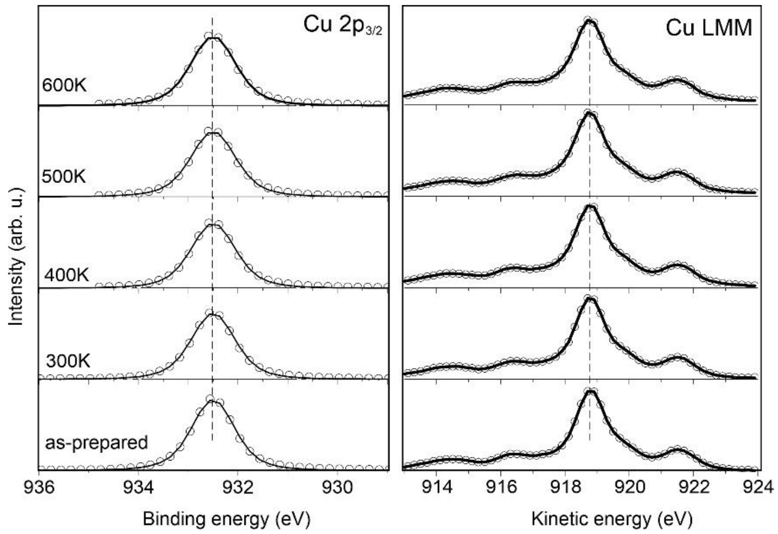

Figure S1. Cu $2p_{3/2}$ core level and Cu LMM Auger lines in XPS spectra of the Ga-Cu(111) surface shown in Figure 2 in the main text after sequential exposure to 10^{-6} mbar of O_2 for 15 min at temperatures (as indicated) increased stepwise from 300 to 600 K. All spectra were measured in UHV at room temperature.

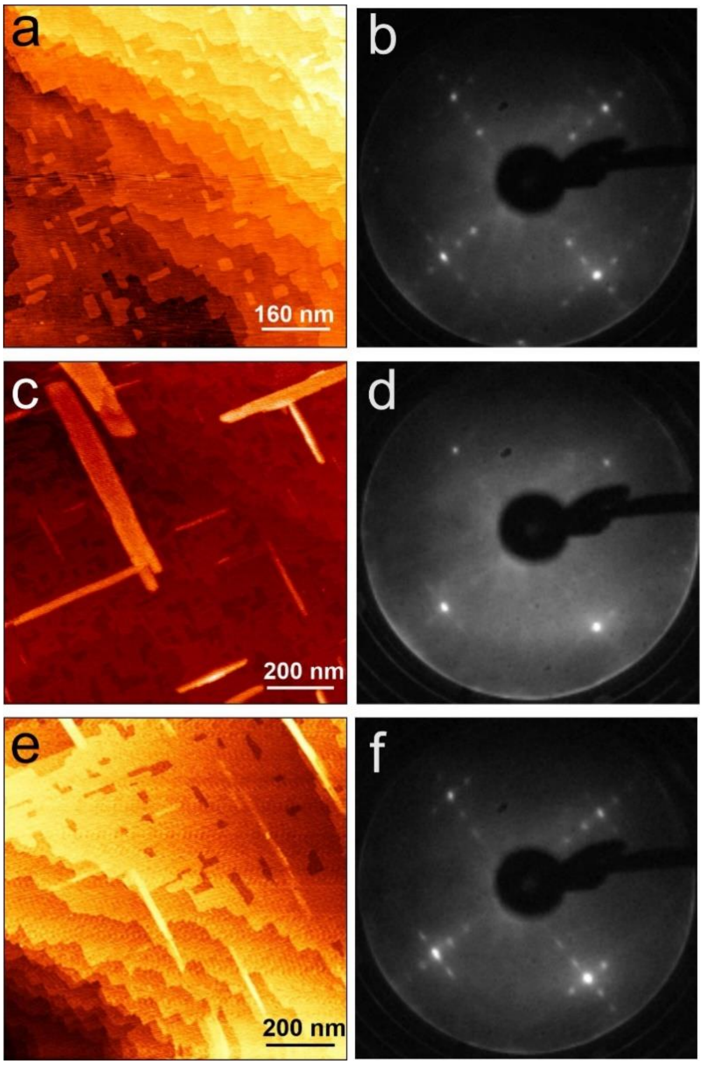

Figure S2. STM and LEED results illustrating the Ga coverage dependence for the formation of the Ga(1×5)-Cu(001) structure. The sample imaged by STM in (a) was prepared by gradual Ga deposition at 300 K until it showed a sharp (1×5) LEED pattern (b). Then the Ga coverage was increased by a factor of two. The surface becomes additionally covered by large deposits several hundreds of nanometers long, up to 100 nm wide, and about 1.5 nm high (c). As a result, the corresponding (1×5) pattern is considerably attenuated (d). However, after UHV annealing of this sample at 600 K for 15 min, the (1×5) structure is restored (f), which is accompanied by the disappearance of the large surface deposits (e).

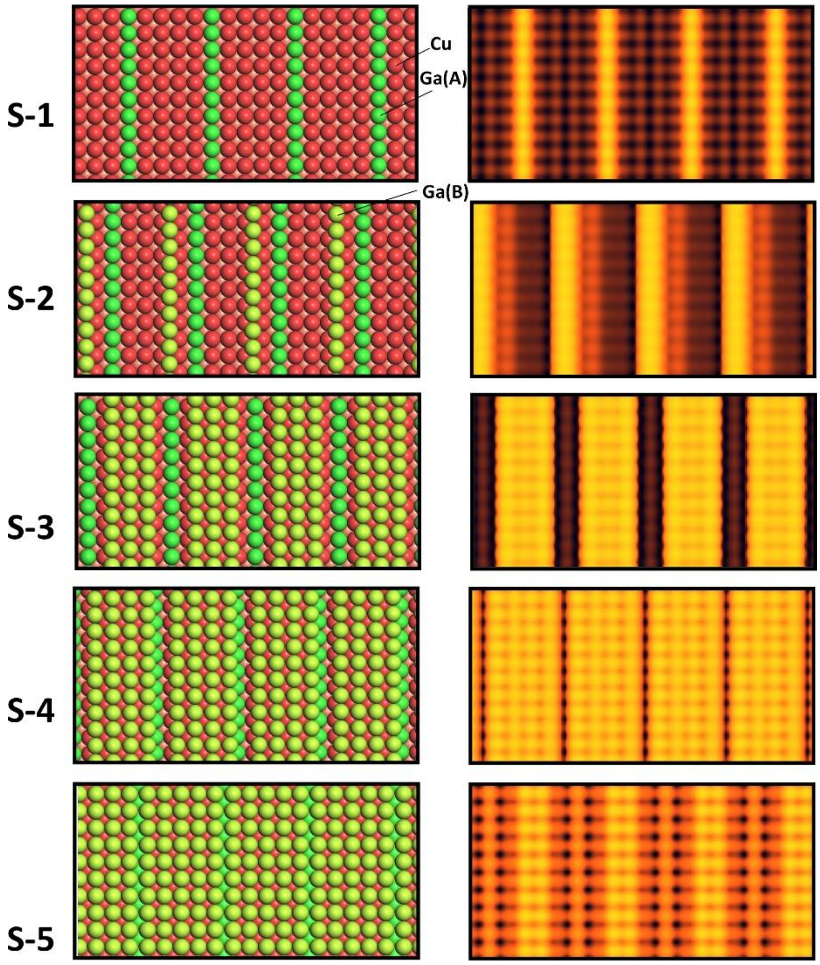

Figure S3. Top views of the atomic structures denoted S-1 – S-5 in the phase diagram presented in Figure 9a, and their simulated STM images in the 0 – 0.2 eV energy range below the Fermi level.

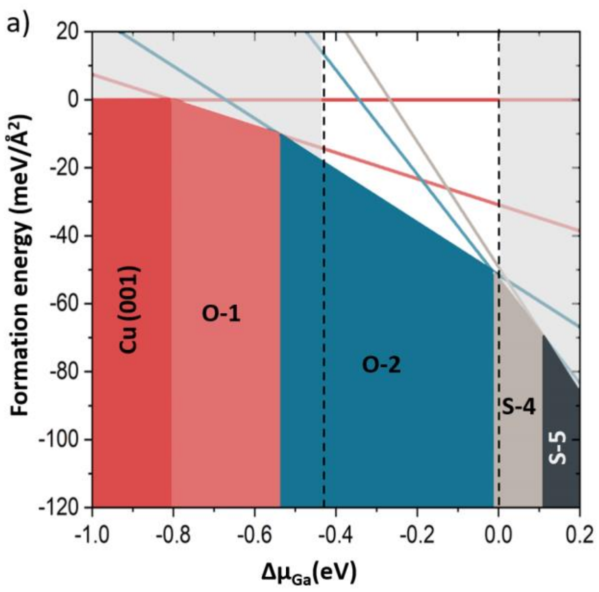

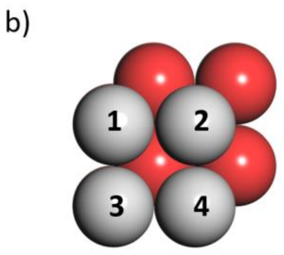

c)

Name	1	2	3	4	γ_{form} (meV/Å²)
**O-1**	Cu	Cu	Cu	Ga	-30.9
**O-2**	Cu	Ga	Ga	Cu	-51.5
**O-3**	Cu	Ga	Ga		-46.9
**O-4**	Ga	Ga	Ga		-47.5

Figure S4. a) Modified phase diagram including (2×2) and c(2×2) structures in addition to (1×5) structures on Cu(001); b) atom labelling in the top layer; c) the formation energies calculated at $\Delta\mu_{Cu} = \Delta\mu_{Ga} = 0$.

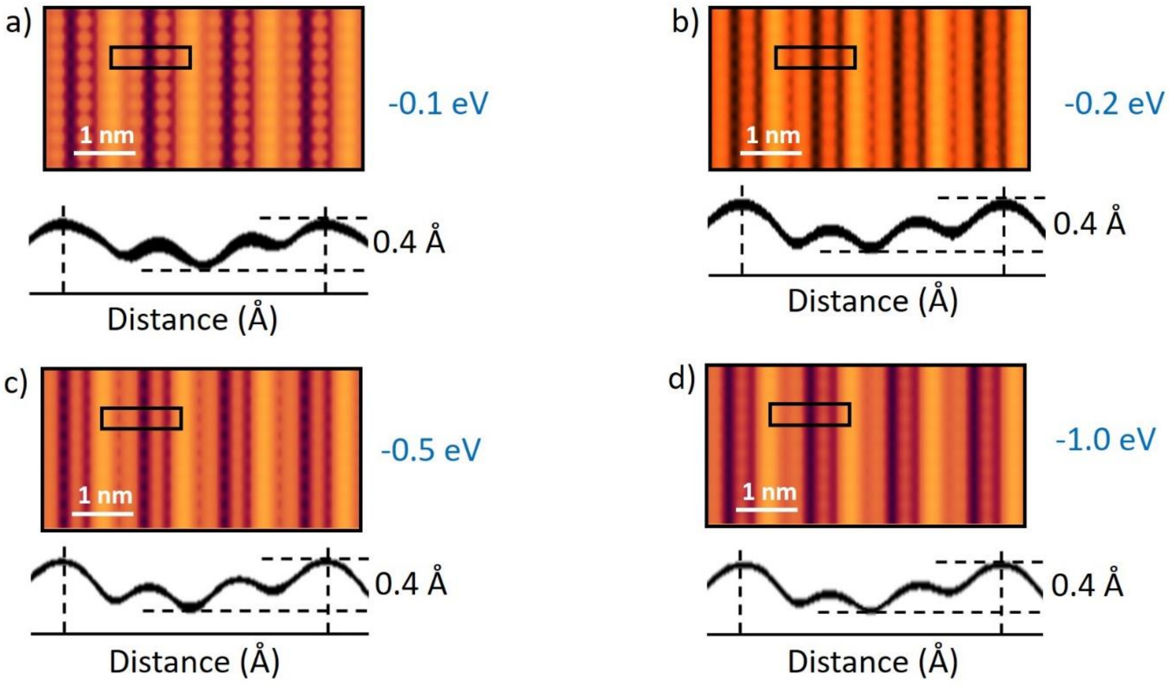

Figure S5. (a-d) Simulated STM images for the most stable Ga(1×5)-Cu(001) structure (i.e., Ga_3Cu_3/Cu_5; the atomic model is shown in Fig. 10b) obtained for several different energy ranges below the Fermi level as indicated. The corrugation profiles within the (1×5) unit cell are shown below the images.

Table S1. Formation energy of the Cu_nGa_m(√3 × √3)R30°-Cu(111) surfaces calculated at $\Delta\mu_{Cu} = \Delta\mu_{Ga} = 0$. Structures marked by (*) do not follow the (√3 × √3)R30° symmetry and are only shown for comparison. Bader charge analysis for the most stable structure is shown below the table.

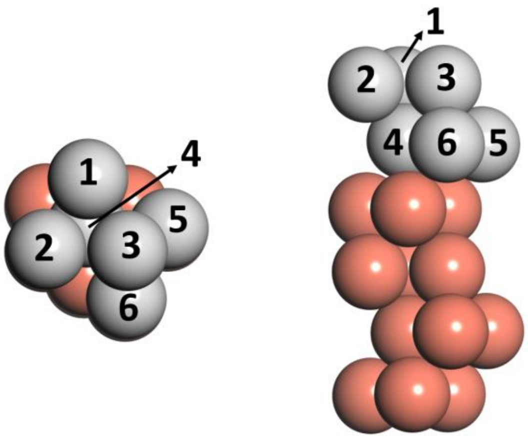

top view **side view**

Surface layer			Subsurface layer			γ_{form} (meV/ Å^2)
**1**	**2**	**3**	**4**	**5**	**6**	
Cu	Ga	Ga	Ga	Cu	Cu	-79.1
Cu	Cu	Ga	Ga	Cu	Cu	-72.4
Cu	Ga	Ga	Cu	Cu	Cu	-59.2
Cu	Cu	Ga	Ga	Cu	Ga	-57.7
Cu	Cu	Ga	Cu	Cu	Cu	-51.9
Ga	Ga		Cu	Cu	Cu	-46.1
Cu	Ga		Cu	Ga	Cu	-35.4
Cu	Cu	Cu	Cu	Ga	Cu	-21.2
Ga	Ga	Ga	Cu	Cu	Cu	-18.5*
Cu	Ga		Cu	Cu	Cu	-11.1
Ga			Cu	Cu	Cu	-4.6
			Cu	Cu	Cu	0.0*
Cu	Cu	Cu	Cu	Ga	Ga	0.4
Cu			Cu	Ga	Cu	12.8
Cu	Cu		Cu	Cu	Ga	22.1
Cu	Cu		Cu	Ga	Ga	39.1
Cu	Cu		Cu	Cu	Cu	46.1
Cu	Cu	Cu	Ga	Ga	Ga	47.7

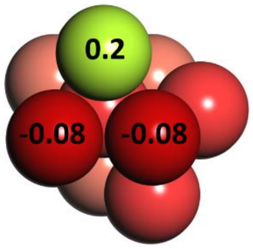

Table S2. Formation energy of the Cu_nGa_m layer (n + m ≤ 5) calculated at $\Delta\mu_{Cu} = \Delta\mu_{Ga} = 0$. The Ga and Cu atoms in the top layer of the Cu(001)-(1 × 5) slab are labelled in the scheme below. Structures marked by (*) do not follow the (1 × 5) symmetry and are only shown for comparison.

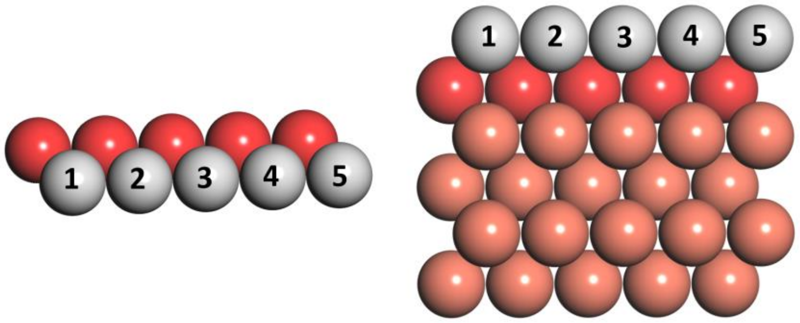

top view **side view**

1	2	3	4	5	γ_{form} (meV/ Å^2)	1	2	3	4	5	γ_{form} (meV/ Å^2)
Ga	Ga	Ga	Ga		-47.2	Ga	Cu	Cu	Cu	Cu	-16.0
	Ga	Ga	Ga	Cu	-46.0		Cu	Ga	Ga	Cu	-15.9
Ga	Ga	Ga	Ga	Ga	-44.8*	Ga					-13.0
Ga	Ga	Cu	Ga	Cu	-42.5			Cu	Cu	Ga	-10.3
Ga	Ga		Ga	Cu	-40.8	Ga				Cu	-9.7
	Ga	Ga	Ga		-37.9	Ga		Cu	Cu	Cu	-9.6
Ga	Ga		Ga		-37.6	Cu	Ga	Cu	Cu		-5.8
Ga	Ga	Cu	Cu	Ga	-37.2			Cu	Ga	Cu	-5.5
Ga	Cu	Cu	Ga	Cu	-32.8	Cu		Ga			-4.9
Cu	Ga	Ga	Ga		-31.8	Ga		Cu			-4.9
		Ga	Cu	Ga	-29.2	Cu	Cu		Ga		-2.7
Cu	Ga		Ga	Cu	-29.0	Cu		Ga	Cu		-1.1
Cu	Ga	Ga	Cu	Cu	-27.1	Cu	Ga		Cu		-1.0
		Ga	Ga		-26.6						0.0*
Cu	Ga		Cu	Ga	-25.3	Cu	Cu	Cu	Cu	Cu	1.0*
Ga		Ga			-25.2				Cu		7.3
Ga	Cu	Cu		Ga	-22.1		Cu	Cu			9.1
Ga	Ga			Cu	-21.6	Cu	Cu	Cu			9.6
Cu		Ga		Ga	-20.5		Cu	Cu	Cu	Cu	10.0
Ga		Cu		Ga	-17.3		Cu		Cu		15.9

Table S3. Formation energy of the Cu_nGa_m layer (n + m ≤ 5) calculated at $\Delta\mu_{Cu} = \Delta\mu_{Ga} = 0$. The Ga and Cu atoms in the surface and subsurface layers of the Cu(001)-(1 × 5) slab are labelled in the scheme below.

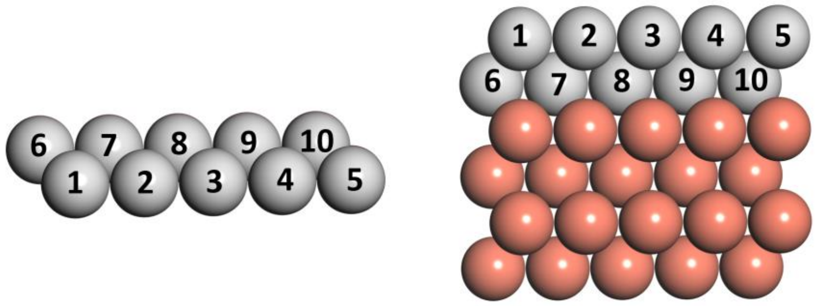

top view **side view**

Surface layer					Subsurface layer					γ_{form} (meV/ Å ²)
**1**	**2**	**3**	**4**	**5**	**6**	**7**	**8**	**9**	**10**	
Ga		Ga	Ga	Ga	Ga	Cu	Cu	Cu	Cu	-52.6
Ga		Ga	Ga	Ga	Cu	Cu	Cu	Ga	Cu	-51.9
Ga	Ga	Ga			Cu	Cu	Cu	Ga	Cu	-50.8
Ga		Ga	Ga	Ga	Cu	Cu	Ga	Cu	Cu	-50.3
Ga	Ga	Ga	Ga	Ga	Ga	Cu	Cu	Cu	Cu	-49.2
Cu	Ga	Ga	Ga	Ga	Ga	Cu	Cu	Cu	Cu	-48.7
Cu	Ga	Ga	Ga	Ga	Cu	Cu	Ga	Cu	Cu	-48.6
Cu	Ga	Ga	Ga	Ga	Cu	Cu	Cu	Ga	Cu	-47.9
Ga	Cu	Ga	Ga	Cu	Cu	Cu	Cu	Ga	Cu	-46.4
Ga	Ga	Ga			Cu	Cu	Ga	Cu	Cu	-45.2
Ga	Cu	Ga	Ga	Cu	Cu	Cu	Ga	Cu	Cu	-44.9
Ga	Cu	Ga	Ga	Cu	Ga	Cu	Cu	Cu	Cu	-44.3
		Ga	Cu	Ga	Ga	Cu	Cu	Cu	Cu	-43.5
Ga	Cu	Cu	Ga	Ga	Cu	Ga	Cu	Cu	Cu	-41.9
Ga	Ga	Ga			Cu	Ga	Cu	Cu	Cu	-41.7
Ga	Cu	Cu	Ga	Ga	Ga	Cu	Cu	Cu	Cu	-40.6
Ga	Cu	Cu	Ga	Ga	Cu	Cu	Cu	Cu	Ga	-40.4
Cu	Ga	Cu		Ga	Cu	Cu	Ga	Cu	Cu	-38.8
Cu	Cu	Ga	Cu	Ga	Ga	Cu	Cu	Cu	Cu	-37.7
Ga		Ga			Cu	Cu	Cu	Ga	Cu	-37.3
Ga			Cu	Ga	Cu	Ga	Cu	Cu	Cu	-36.1
Ga			Cu	Ga	Cu	Cu	Ga	Cu	Cu	-35.9
		Ga	Cu	Ga	Cu	Cu	Cu	Cu	Ga	-35.7
Cu	Cu	Ga	Cu	Ga	Cu	Cu	Cu	Cu	Ga	-35
Cu	Cu	Ga		Ga	Cu	Cu	Cu	Ga	Cu	-34.9
Cu	Cu	Ga	Cu	Ga	Cu	Cu	Cu	Ga	Cu	-34.8
Cu	Cu		Ga	Ga	Cu	Ga	Cu	Cu	Cu	-34.7
Cu	Cu	Ga		Ga	Ga	Cu	Cu	Cu	Cu	-34.4
Ga		Ga			Cu	Cu	Ga	Cu	Cu	-32
Ga		Ga			Cu	Cu	Cu	Cu	Ga	-31.9
		Ga	Cu	Ga	Cu	Cu	Ga	Cu	Cu	-31.5

Table S3 (continued).

**Surface layer**					**Subsurface layer**					
**1**	**2**	**3**	**4**	**5**	**6**	**7**	**8**	**9**	**10**	**γ_{form} (meV/ Å^2)**
Ga		Ga			Ga	Cu	Cu	Cu	Cu	-31.3
	Cu	Ga	Cu	Ga	Cu	Cu	Cu	Cu	Ga	-31.2
Cu	Ga	Cu		Ga	Cu	Cu	Cu	Ga	Cu	-31.1
Cu	Cu	Ga	Ga	Cu	Cu	Cu	Cu	Ga	Cu	-30.2
				Ga	Cu	Ga	Cu	Cu	Cu	-29.6
Cu		Cu	Ga	Ga	Cu	Ga	Cu	Cu	Cu	-29
Cu	Cu	Ga	Ga	Cu	Cu	Cu	Ga	Cu	Cu	-28.9
Ga			Cu	Ga	Ga	Cu	Cu	Cu	Cu	-28.8
	Cu		Ga	Ga	Cu	Ga	Cu	Cu	Cu	-28.1
Cu	Ga	Cu		Ga	Ga	Cu	Cu	Cu	Cu	-26.7
				Ga	Ga	Cu	Cu	Cu	Cu	-27.9
	Cu	Ga	Cu	Ga	Cu	Ga	Cu	Cu	Cu	-26.5
Cu	Ga	Cu		Ga	Cu	Ga	Cu	Cu	Cu	-26.4
Cu	Cu		Ga	Ga	Ga	Cu	Cu	Cu	Cu	-25.6
	Cu		Ga	Ga	Cu	Cu	Cu	Cu	Ga	-25.5
Ga			Cu	Ga	Cu	Cu	Cu	Ga	Cu	-25.3
Cu				Ga	Cu	Ga	Cu	Cu	Cu	-25.2
Cu	Cu		Ga	Ga	Cu	Cu	Cu	Cu	Ga	-24.8
Cu			Cu	Ga	Cu	Ga	Cu	Cu	Cu	-20.6
				Ga	Cu	Cu	Cu	Cu	Ga	-20.5
				Ga	Cu	Cu	Cu	Ga	Cu	-20.4
Cu	Ga	Cu		Cu	Cu	Cu	Ga	Cu	Cu	-19.8
Cu	Ga	Cu		Cu	Cu	Cu	Cu	Ga	Cu	-19.5
	Cu	Ga	Cu	Cu	Cu	Cu	Cu	Cu	Ga	-19.4
Cu			Cu	Ga	Cu	Cu	Ga	Cu	Cu	-19.3
	Cu		Ga	Ga	Cu	Cu	Cu	Ga	Cu	-19.1
Cu		Cu	Ga	Ga	Cu	Cu	Ga	Cu	Cu	-18.9
Ga		Cu			Cu	Cu	Cu	Ga	Cu	-18.6
Ga	Cu	Cu	Cu	Cu	Ga	Cu	Cu	Cu	Cu	-18.2
Cu		Cu	Ga	Ga	Cu	Cu	Cu	Ga	Cu	-17.3
	Cu		Ga		Ga	Cu	Cu	Cu	Cu	-16.2
Cu			Ga	Cu	Cu	Cu	Ga	Cu	Cu	-16.1
					Ga	Cu	Cu	Cu	Cu	-15.9
Cu				Ga	Cu	Cu	Cu	Ga	Cu	-15.6
			Cu	Ga	Cu	Cu	Cu	Cu	Ga	-15.5
Cu			Ga	Cu	Cu	Cu	Cu	Cu	Ga	-14.4
Cu	Cu		Ga		Cu	Ga	Cu	Cu	Cu	-13.7
Ga		Cu			Ga	Cu	Cu	Cu	Cu	-12.0
			Cu	Ga	Cu	Cu	Cu	Ga	Cu	-11.5
	Cu		Ga		Cu	Cu	Cu	Ga	Cu	-11.4
Ga			Cu	Cu	Cu	Cu	Cu	Cu	Ga	-11.3
Cu			Ga	Cu	Cu	Cu	Cu	Ga	Cu	-11.2
Cu	Cu		Ga		Cu	Cu	Cu	Ga	Cu	-9.4
Cu	Ga	Cu		Cu	Cu	Cu	Cu	Cu	Ga	-9.2

Table S3 (continued).

**Surface layer**					**Subsurface layer**					
**1**	**2**	**3**	**4**	**5**	**6**	**7**	**8**	**9**	**10**	**γ_{form} (meV/ Å^2)**
Cu	Ga	Cu		Cu	Cu	Ga	Cu	Cu	Cu	-7.6
	Cu	Ga	Cu	Cu	Cu	Ga	Cu	Cu	Cu	-7.5
Cu	Ga	Cu		Cu	Ga	Cu	Cu	Cu	Cu	-7
	Cu	Ga	Cu	Cu	Cu	Cu	Ga	Cu	Cu	-6.9
Cu			Cu	Ga	Cu	Cu	Cu	Ga	Cu	-6.6
		Cu	Cu		Cu	Cu	Cu	Cu	Ga	-5.8
Cu	Cu	Cu			Cu	Cu	Cu	Ga	Cu	-5.4
Cu	Cu	Cu	Cu	Cu	Ga	Cu	Cu	Cu	Cu	-4.9
Cu	Cu		Ga		Ga	Cu	Cu	Cu	Cu	-4.8
Cu	Cu	Cu			Cu	Cu	Ga	Cu	Cu	-4.2
		Cu	Cu		Cu	Cu	Cu	Ga	Cu	-3.9
Cu	Cu	Cu		Cu	Cu	Cu	Ga	Cu	Cu	-3
Cu	Cu	Cu		Cu	Cu	Cu	Cu	Ga	Cu	-3
Cu		Cu			Cu	Cu	Cu	Ga	Cu	0.4
Cu		Cu			Cu	Cu	Ga	Cu	Cu	4.2
Cu		Cu			Cu	Cu	Cu	Cu	Ga	4.2
Cu		Cu			Ga	Cu	Cu	Cu	Cu	4.4
	Cu	Cu		Cu	Cu	Cu	Ga	Cu	Cu	5.1
Cu	Cu	Cu		Cu	Ga	Cu	Cu	Cu	Cu	5.3
Cu	Cu	Cu		Cu	Cu	Cu	Cu	Cu	Ga	5.6
		Cu	Cu		Cu	Cu	Ga	Cu	Cu	5.7
	Cu	Cu		Cu	Cu	Cu	Cu	Cu	Ga	6.6
	Cu	Cu		Cu	Cu	Ga	Cu	Cu	Cu	14.6

Table S4. Formation energy of all non-commensurate Cu_nGa_m layers (n + m = 6) calculated at $\Delta\mu_{Cu} = \Delta\mu_{Ga} = 0$. The Ga and Cu atoms within the top layer over the Cu(001)-(1 × 5) unit cell are labelled following the scheme below. Bader charge analysis for the most stable structure is shown below this table.

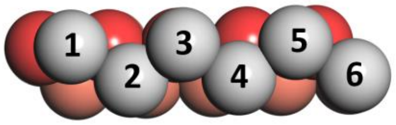

top view

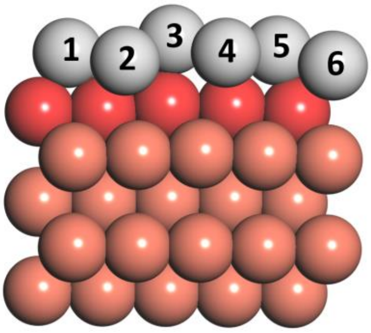

side view

**1**	**2**	**3**	**4**	**5**	**6**	**γ_{form} (meV/ Å ^{2})**
Ga	Cu	Ga	Cu	Ga	Cu	-56
Cu	Cu	Cu	Ga	Cu	Ga	-33.6
Cu	Cu	Ga	Cu	Cu	Ga	-28.5
Cu	Ga	Cu	Ga	Ga	Ga	-26.8
Cu	Cu	Cu	Cu	Ga	Ga	-18.6
Cu	Ga	Ga	Ga	Ga	Ga	-15.8
Cu	Cu	Cu	Cu	Cu	Ga	-12.2
Ga	Ga	Ga	Ga	Ga	Ga	2
Cu	Cu	Cu	Cu	Cu	Cu	11.9

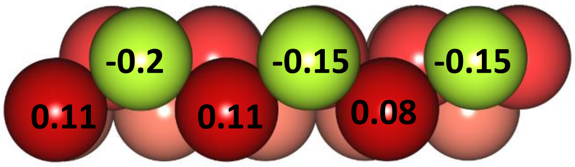

Supplement: Supplementary file 1 — jp3c05711_si_001.pdf [file jp3c05711_si_001.pdf]
